# Sarcopenia in Older Adults with Hematologic Malignancies: A Comprehensive Review of Epidemiology, Prognosis, and Interventions

**DOI:** 10.3390/cancers18030503

**Published:** 2026-02-03

**Authors:** Samuel J. Yates, Nadine H. Abdallah, Konstantinos Christofyllakis, Brandon N. VanderVeen, Timothy S. Pardee, Denise K. Houston, Stephen B. Kritchevsky, Heidi D. Klepin

**Affiliations:** 1 Department of Internal Medicine, Section on Hematology and Oncology, University of Chicago, Chicago, IL 60637, USA; 2 Department of Oncology, Mayo Clinic, Rochester, MN 55905, USA; abdallah.nadine@mayo.edu; 3 Department of Hematology and Oncology, Clinical Immunology and Rheumatology, Saarland University Medical Center, D-66421 Homburg, Germany; konstantinos.christofyllakis@uks.eu; 4 Department of Cancer Biology, Wake Forest University School of Medicine, Winston-Salem, NC 27157, USA; brandon.vanderveen@advocatehealth.org (B.N.V.); timothy.pardee@wfusm.edu (T.S.P.); 5 Department of Cancer Medicine, Atrium Health Wake Forest Baptist Comprehensive Cancer Center, Winston-Salem, NC 27157, USA; heidi.klepin@wfusm.edu; 6 Department of Internal Medicine, Section on Gerontology and Geriatric Medicine, Wake Forest University School of Medicine, Winston-Salem, NC 27157, USA; denise.houston@wfusm.edu (D.K.H.); stephen.kritchevsky@advocatehealth.org (S.B.K.)

**Keywords:** sarcopenia, hematologic malignancy, geriatric, cachexia, malnutrition

## Abstract

Hematologic malignancies, including leukemias, lymphomas, and multiple myeloma, are diseases of older adults (age ≥ 60 years). Treatment decisions in older adults with cancer are heavily influenced by patient fitness: the ability of an individual patient to tolerate the off-target effects of anti-cancer therapy. Sarcopenia, defined as low muscle mass and strength, is increasingly recognized as an important marker of fitness. Furthermore, sarcopenia is known to be modifiable with nutrition and exercise interventions in older adults without cancer, but such evidence is limited for patients with cancer. Much of the literature on sarcopenia in older adults with cancer has focused on patients with solid tumors. However, there is an increasing body of literature on the impact of sarcopenia in older adults with hematologic malignancies. In this comprehensive review, we attempt to (i) describe the most up-to-date diagnostic criteria and diagnostic approach to sarcopenia, (ii) summarize the prognostic impact of sarcopenia among older adults with hematologic malignancies, (iii) discuss the proposed mechanisms of sarcopenia and its pathogenesis, (iv) review the evidence for interventions targeting sarcopenia, and (v) provide future directions for the advancement of sarcopenia management among older adults with hematologic malignancies.

## Simple Summary

Blood cancers include leukemias, lymphomas, and multiple myeloma. Blood cancers are diseases of older adults (age ≥ 60 years). As the human body ages, the amount of muscle and the strength of that muscle begin to decline. When muscle quantity and muscle strength are low enough, this is called sarcopenia. Over the past twenty years, the field of oncology has recognized that patients with cancer are affected with sarcopenia more often than those who do not have cancer. Furthermore, sarcopenia puts cancer patients at risk for tolerating cancer treatments poorly and passing away from their cancer earlier than those without sarcopenia. Much of what is published on the topic of sarcopenia in older adults with cancer has focused on cancers of the lung, breast, and colon. To fill this gap in knowledge, we provide a comprehensive review of the available information on sarcopenia among older adults with blood cancers. We describe the definition of sarcopenia, its prevalence in different blood cancers, interventions for sarcopenia, and future directions for the care of older adults with blood cancers who have sarcopenia.

## 1. Introduction

**Figure 1:**
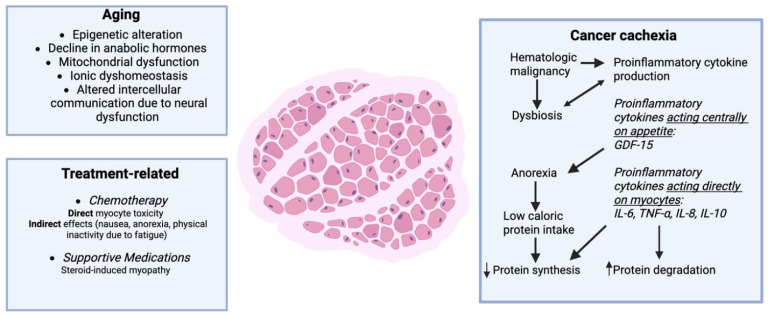
Mechanisms of sarcopenia development among older adults with hematologic malignancies. Created in BioRender. Yates, S. (2026) https://BioRender.com/r24ikop.

Sarcopenia, derived from the Greek words *sarx* for flesh and *penia* for loss, is defined as low muscle mass and strength. Sarcopenia was initially described in 1989 as an aging-related condition, with a linear decline in muscle mass and strength occurring as early as the fourth decade of life [1,2,3]. Of the 12 Hallmarks of Aging [4], current data suggest epigenetic alteration, mitochondrial dysfunction, and altered intercellular communication are major drivers of skeletal muscle aging [5]. Global prevalence and incidence estimates of sarcopenia are widely debated due to the heterogeneity in diagnostic criteria utilized. A conservative estimate of 5–10% prevalence of sarcopenia in the older adult population is generally accepted [6]. The clinical significance of sarcopenia in older adults (aged ≥ 60 years) is well described. Multiple studies have shown that sarcopenia is associated with an increased likelihood of falls, fractures, physical disability, and all-cause mortality [7]. In 2004, Shen et al. described the methodology for estimating total-body skeletal muscle and adipose tissue from a single abdominal computed tomography (CT) cross-sectional image, typically at the third lumbar spinal level [8]. The following year, this methodology was validated in patients with cancer. In 2007, the independent prognostic impact of sarcopenia in patients receiving chemotherapy was first described [9,10]. Since that time, hundreds of studies have detailed the prognostic value of sarcopenia in older adults with cancer [11]. Cancer compounds the age-related [5] decline in muscle mass and strength by inducing cachexia [12], promoting physical inactivity and muscle atrophy, and creating a chronic proinflammatory state. Taken together, this results in a net catabolic state leading to muscle breakdown (Figure 1) [13].

Additionally, anti-cancer therapies contribute to both indirect (anorexia, nausea, proinflammatory state) and direct muscle breakdown via pathways that upregulate proteasome activity and induce mitochondrial dysfunction [13,14,15,16,17]. Historically, studies on sarcopenia in the oncologic setting have focused on patients with solid tumors such as head and neck cancers and gastrointestinal cancers [11]. CT imaging is the standard of care for monitoring disease response and surveillance for disease recurrence in most solid tumors; therefore, body composition data are readily available for retrospective research use. In contrast, sarcopenia among patients with hematologic malignancies is understudied.

Most hematologic malignancies, encompassing leukemias, lymphomas, and multiple myeloma (MM), are considered diseases of older adults [18]. Though heterogenous in disease biology, natural history, and treatment options, hematologic malignancies confer significant morbidity and mortality in older adults. Life-long treatment is required for many chronic, indolent blood cancers, such as chronic lymphocytic leukemia (CLL) and follicular lymphoma (FL), while acute, aggressive blood cancers, such as acute myeloid leukemia (AML) and diffuse large B-cell lymphoma (DLBCL), have high early death rates and poor overall survival (OS) [19]. Unlike in the pediatric, adolescent, and young adult oncology populations, treatment decision-making in older adults is heavily influenced by patient fitness [20]. Older patients are generally categorized as fit, intermediate fit, and frail corresponding with the intensity of therapies patients are offered. For example, ALL in children and adolescents is treated with high-dose, multidrug regimens administered over a 2–3-year period. These regimens have incredibly high cure rates in children and adolescents but are associated with high morbidity and mortality rates in older adults, often prohibiting use. Unfortunately, the tradeoff of a less intensive regimen often comes with lower cure rates in older adults. Therefore, optimally defining fitness is critical as it facilitates choosing a regimen that both maximizes the chance of cure while minimizing toxicities. Furthermore, fitness is not static; changes occur as the patient progresses through treatment, necessitating a dynamic approach to fitness evaluation [21,22,23,24].

Fitness was historically defined using a combination of age and the physician-assigned performance status, which amounts to little more than “the eye-ball test” [25]. Over the past two decades, collaborative efforts between the oncologic and geriatric societies have established the comprehensive geriatric assessment (CGA) as the standard-of-care tool for assessing fitness among older adults with cancer [26,27,28,29]. A CGA holistically assesses domains of an older adult’s health that are not captured by age and performance status alone [30,31,32]. While the CGA includes measures of nutritional status and physical function, the current definition does not include measures of sarcopenia

The present literature in hematologic malignancies lacks an updated review on the topic of sarcopenia. Particularly, ambiguity remains on the current definition of sarcopenia, the differences between cachexia and sarcopenia, and potential interventions to treat sarcopenia. In this comprehensive review, we attempt to (i) describe the most up-to-date diagnostic criteria and diagnostic approach to sarcopenia, (ii) summarize the evidence on the prognostic impact of sarcopenia among older adults with hematologic malignancies, (iii) discuss the proposed mechanisms of sarcopenia development and pathogenesis, (iv) review the evidence for interventions targeting sarcopenia, and (v) provide future directions for the advancement of sarcopenia management among older adults with hematologic malignancies.

## 2. Sarcopenia: Diagnostic Approach

The diagnostic criteria for sarcopenia have undergone multiple iterations and include recommendations (Table 1) from the Sarcopenia Definitions and Outcomes Consortium (SDOC) [33], Asian Working Group on Sarcopenia (AWGOS) [34], European Working Group on Sarcopenia in Older People (EWGSOP) [35], and the Australian and New Zealand Society for Frailty Research (ANZSSFR) [36]. However, in 2024, a collaborative effort to consolidate these criteria was published by the Global Leadership in Sarcopenia (GLIS) [37]. Using a Delphi Poll method, 20 statements were agreed upon regarding (1) general aspects of sarcopenia, (2) components of sarcopenia, and (3) outcomes of sarcopenia. The requirement for low muscle mass (myopenia) and low muscle strength (dynapenia) was retained in the GLIS criteria. This remains the most up-to-date consensus definition [37]. Many studies in oncology, particularly those predating the GLIS criteria, only utilize myopenia in their definition of sarcopenia. Muscle quality, particularly the degree of fat infiltration in muscle, termed myosteatosis, was narrowly not retained in the GLIS definition. Controversy remains regarding this point in light of data demonstrating the prognostic relevance of myosteatosis in older adults, including in those with cancer.

**Figure 2:**
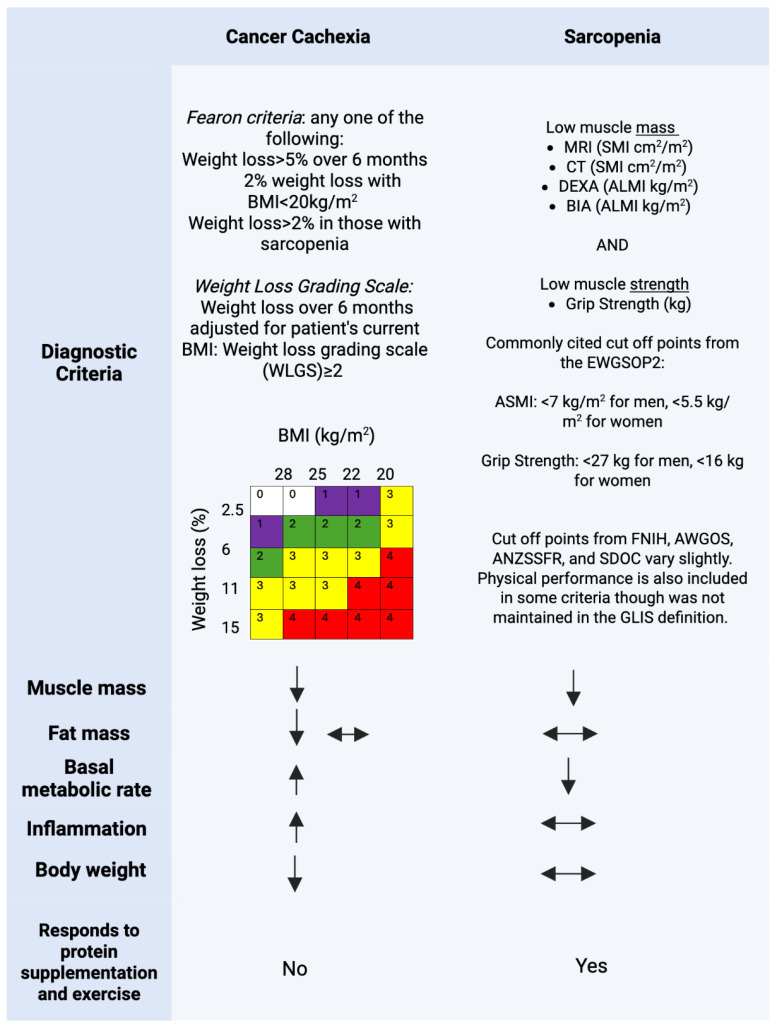
Diagnostic criteria and phenotypic features of sarcopenia and cancer cachexia. For a full list of diagnostic criteria across different international consensus groups, see Table 1. ↑ indicates increase; ↓, decrease; and ↔, unchanged. The presence of 2 arrows indicates that either of the changes in the variable may occur in the syndrome (e.g., cancer cachexia may be accompanied by a decrease or no change in fat mass). ALMI, appendicular lean mass index; ASMI, appendicular skeletal muscle Index; SMI, skeletal muscle index. Created in BioRender. Yates, S. (2026) https://BioRender.com/9sk0rnd.

In the clinical setting, several tools (Figure 2) have been recommended for the measurement of muscle mass and strength.

Grip strength measured using a handheld dynamometer is nearly unanimously used for assessment of muscle strength. To date, no reliable method for measuring whole body muscle mass in a scalable and cost-effective manner is available [6]. CT and MRI imaging, whereby a cross-section is extrapolated to whole body measurement [8], remains the gold-standard. The use of CT and MRI imaging in daily practice has been criticized due to the time needed to obtain the measure, associated costs, and risk of radiation-related adverse effects. In comparison to CT, which utilizes Hounsfield units to directly measure skeletal muscle mass, bioelectrical impedance analysis (BIA) is a technology that estimates skeletal muscle mass from the measurement of impedance of body tissues to a small electric current [38]. BIA estimates body composition using two compartments: fat mass and fat free mass/lean body mass (body water, muscle, and bone). Because there are no solid organs aside from skeletal muscle and bones in the appendicular skeleton, the appendicular lean mass (ALM) from a BIA-measurement has been used as a surrogate of appendicular skeletal muscle mass (ASMM). There are a variety of BIA devices with varying degrees of accuracy that are utilized in oncology clinical practice, a topic beyond the scope of this narrative review but nicely summarized elsewhere [39]. Dual-energy X-ray absorptiometry (DEXA) utilizes similar principles as BIA and has been used for many decades. Sarcopenia screening tools using patient-reported data include the SARC-F and SarcoPRO. SARC-F [40], a five-item questionnaire recommended by the EWGSOP2, is the most widely utilized. In the oncologic setting, the performance of SARC-F has, to date, been poor (sensitivity: 28.9–55.3%; specificity: 68.9–88.9%) [41] and, thus, it is not routinely utilized.

## 3. Sarcopenia and Cancer Cachexia: A Diagnostic Conundrum

Sarcopenia and cancer cachexia are overlapping, yet distinct entities (Figure 2), a distinction that can cause significant confusion for clinicians. Cancer cachexia is a catabolic inflammatory syndrome where complex metabolic changes to the host lead to a loss of total body and skeletal muscle mass. The diagnostic criteria for cancer cachexia [42,43] reflect manifestations of the syndrome rather than a deep understanding of the biologic processes that drive cancer cachexia, which are poorly described to date [12,44,45]. The Fearon Criteria, considered the gold standard, defines cancer cachexia as greater than 5% weight loss in the prior 6 months, or 2–5% weight loss with either a BMI of <20 kg/m^2^ or reduced muscle mass [46]. The Weight Loss Grading Scale, which considers all weight loss in the context of BMI, is also commonly utilized [43]. Older adults with cancer who are affected by cachexia or sarcopenia often phenotypically resemble one another, and both conditions can lead to similar complications. However, despite their overlap, they describe two distinct entities. Sarcopenia requires low muscle strength, while cachexia does not. Cachexia requires weight loss, while sarcopenia may be diagnosed in a patient with unchanged weight. This difference is not simply theoretical; when considering how to intervene on a patient who meets the diagnostic criteria for both these entities, clearly defining which of these conditions predominates may have a significant impact on their clinical improvement. For example, a patient with cancer cachexia who is experiencing sarcopenia secondarily may not experience clinical benefit from protein supplementation and exercise alone. In such a case, concurrently targeting the underlying inflammatory state from cancer cachexia is essential as will be discussed further below (Section 6).

## 4. Sarcopenia in Older Adults with Hematologic Malignancies

In the following section, we will review the available data on sarcopenia in each of the major disease groups: leukemias, lymphomas, and MM. Table 2 summarizes these data by disease group, prevalence, and outcomes assessed. We clearly note whether studies utilized both muscle mass and muscle strength to define sarcopenia or utilized a measure of myopenia alone, in line with the current GLIS criteria.

### 4.1. Sarcopenia Among Older Adults with Leukemia

AML is the most common and lethal acute leukemia in older adults with a median OS of ~1 year [81,82]. AML management begins with chemotherapy initiation to achieve a remission, typically with the combination of a hypomethylating agent and the BCL-2 inhibitor, Venetoclax [82]. Patients either stay on this treatment indefinitely or may proceed to allogeneic stem cell transplant (alloHSCT) which offers a chance of cure. Sarcopenia or myopenia have been evaluated both in those newly diagnosed with AML [24,47,48,49,50,51] and in the setting of alloHSCT (Table 2) [54,56,83,84]. In the newly diagnosed setting, prevalence ranges from 15 to 43% though only two studies [24,51] utilized a measure of both muscle mass and strength in defining sarcopenia (15% and 24%). Furthermore, only one study exclusively assessed older adults [24], while the others had varying numbers of older adults (42–62%). In each of these studies, pre-induction sarcopenia and myopenia were associated with poor OS, including in multivariable analyses controlling for age, disease risk, and performance status. Higher early death rates were also reported in those with sarcopenia. Qin et al [51] additionally aimed to define the change in prevalence of sarcopenia before and after induction chemotherapy, demonstrating significant increases in sarcopenia after induction (15% versus 39%; *p* < 0.001).

The prevalence of myopenia in those proceeding to alloHSCT is even higher than in the newly diagnosed setting. Indeed, in 1130 patients across studies of alloHSCT prevalence of pre-alloHSCT, myopenia ranged from 33.7 to 55% [83]. All studies found a negative impact on non-relapse mortality (NRM) (pooled hazard ratio: 1.70; 95% CI: 1.32–2.18) [83] and OS. Two studies also assessed myopenia at 180 days [85], 1 year, and 2.5 years [84] post-alloHSCT finding an increase in myopenia at each time point. In these studies, multivariable analysis for NRM controlled for performance status, age, and, in one study, CGA [85]. These results suggest myopenia is a unique biomarker of fitness rather than simply reflecting the impact of a patient’s comorbidities, age, or disease severity. Potential mechanisms by which myopenia prior to alloHSCT may be driving higher NRM included higher incidence of high-grade acute graft versus host disease [84] and fatal infections [54] in those with sarcopenia and myopenia. No studies in alloHSCT have utilized a measure of muscle strength to define sarcopenia.

Only single studies have assessed the impact of sarcopenia and myopenia on outcomes in CLL and ALL. In the HOVON 139/GiVE trial of older adults with CLL receiving venetoclax and obinatuzumab, Van der Straten et al. utilized a CGA inclusive of sarcopenia defined by EWGSOP criteria. Sarcopenia occurred in 13% of the cohort at diagnosis and was roughly stable 1-year and 15 months into therapy (10% and 9%, respectively) [21]. The investigators did not report on the association of sarcopenia with any treatment outcomes. In ALL, one retrospective study showed worse survival in those with myopenia. This cohort was skewed heavily to younger adults, making it difficult to extrapolate to an older adult population where treatment regimens are significantly different [57,86,87].

Finally, to date, no studies have described the impact of sarcopenia among patients with myeloproliferative neoplasms (polycythemia vera, essential thrombocythemia, and myelofibrosis), chronic myeloid leukemia, or myelodysplastic syndromes. Given the breadth of literature on the impact of frailty in these conditions [20,88,89], and the significant overlap between frailty and sarcopenia, further investigation is warranted.

### 4.2. Sarcopenia Among Older Adults with Lymphomas

Lymphomas are broadly categorized into Hodgkin’s (HL) and non-Hodgkin’s lymphomas (nHL). Sarcopenia and myopenia have primarily been described in DLBCL, the most common and aggressive nHL. Approximately 55% of patients with DLBCL present with myopenia at diagnosis, rising to 63% in older adults (Table 2) [58,59]. Lanic et al. were the first to demonstrate that myopenia is an independent adverse prognostic factor for OS. However, in a larger, prospective multicenter study by the same researchers, myopenia did not independently predict worse survival [60]. In a meta-analysis of 12 studies including 2345 patients of all ages with DLBCL, myopenia was associated with poorer OS and progression-free survival (PFS), as well as lower rates of treatment completion and complete remission [90]. Indolent lymphomas are a subgroup of nHL characterized by a less aggressive course, often without need for immediate treatment. Chu et al. examined the prognostic role of myopenia in patients with newly diagnosed follicular lymphoma; myopenia was prevalent (40.7%) but not associated with OS or PFS [91]. In mantle cell lymphoma, a high prevalence (60%) of myopenia, and an association with shorter PFS, have been reported [63]. HL is uncommon among older adults, hence studies on sarcopenia and myopenia are rare. In a multicenter study of older adults treated for newly diagnosed HL, 73% were found to be myopenic, though with no impact on clinical outcomes [62].

The treatment of relapsed/refractory lymphomas utilizes cellular therapies including autologous hematopoietic stem cell transplantation (autoHSCT), bispecific antibodies, and chimeric antigen receptor T-cell therapy (CAR-T). In a study of 320 patients with HL and nHL undergoing autoHSCT, myopenic patients aged 18–78 years (median age 53.3 years) had worse OS, increased length of stay, and higher 30-day unplanned readmission [64]. Similarly, in a meta-analysis examining the impact of myopenia in patients with lymphoma undergoing autoHSCT, 44.2% of patients were myopenic prior to autoHSCT. Myopenia was associated with increased mortality (HR 1.92, *p* < 0.001) [92]. Only two retrospective single center studies evaluating myopenia in CAR-T are available [66,67]. The median age ranged from 64 to 66 years with ~50% of patients myopenic prior to CAR-T. Myopenia was associated with higher NRM, worse PFS, and worse OS in these studies [66,67]. To date, no studies have described the impact of sarcopenia or myopenia among patients receiving bispecific antibody therapy for lymphoma.

In summary, most studies of sarcopenia in patients with lymphoma have only evaluated myopenia. Dedicated studies utilizing the GLIS criteria for sarcopenia are needed. However, available data suggests myopenia is associated with worse survival in most treatment modalities, particularly in those with DLBCL. Current data from HL and indolent lymphomas are limited but suggest myopenia may not be clinically relevant.

### 4.3. Sarcopenia Among Older Adults with Multiple Myeloma

MM is an incurable plasma cell dyscrasia that disproportionately affects older adults (median age at diagnosis ≥ 69 years) [93]. Consequently, patients with MM are at increased risk of aging-related conditions, including frailty and sarcopenia. Contemporary frontline therapy typically consists of triplet or quadruplet regimens incorporating proteasome inhibitors, immunomodulatory drugs, corticosteroids, and CD38-directed monoclonal antibodies [94]. Following 4–6 cycles of induction, patients deemed transplant-eligible may undergo consolidation with high-dose chemotherapy and autoHSCT. The criteria for transplant eligibility vary across clinical trials and practice settings, but are largely influenced by age, comorbidities, and functional status [95]. While frailty has been established as an independent predictor of inferior outcomes in MM [31], the prognostic significance of sarcopenia remains less well defined. Several studies have evaluated sarcopenia, myopenia, and myosteatosis in newly diagnosed MM, mostly using retrospective analyses of muscle mass and/or quality assessed from CT imaging. Prevalence estimates vary widely—from 20% [74] to over 80% [71]—largely due to heterogeneity in definitions and cutoffs. Similarly, reported associations between sarcopenia and myopenia and treatment outcomes have been inconsistent. When defined solely by myopenia, a significant association with transplant-related outcomes or survival was observed in a minority of studies [74,76], while most found no effect [70,71,72,73,75,77,78]. In contrast, myosteatosis has more consistently been correlated with adverse treatment outcomes, including transplant-related morbidity and survival. In a retrospective study of 341 newly diagnosed MM patients, Abdallah et al. reported that myosteatosis was associated with more aggressive disease features and independently predicted OS, whereas myopenia alone was not [70]. Similarly, Diallo et al. found that 51% of 226 patients had myosteatosis, which correlated with frailty, higher disease stage, and worse PFS and OS [77]. In both studies, patients with myosteatosis were less likely to proceed to autoHSCT, suggesting an association with perceived fitness for transplant. In another study of 142 patients with MM, myosteatosis was present in 51% and associated with increased risk of early post-transplant cardiovascular complications but not OS [69]. Taken together, these findings suggest that muscle quality may offer prognostic value beyond myopenia alone in those transplant-eligible at diagnosis. Nonetheless, the prognostic relevance of sarcopenia appears to be context-dependent, varying across age groups and treatment settings. For example, in the HOVON123 trial, which enrolled older (≥75 years), non-transplant-eligible patients (*n* = 220), myopenia was associated with early treatment discontinuation and inferior survival, whereas sarcopenia, utilizing handgrip strength and skeletal muscle index (SMI), was not predictive [80]. Furthermore, a composite definition combining low mass, strength, and function did not provide additional prognostic value in this population.

Unlike the newly diagnosed setting, the impact of sarcopenia in relapsed/refractory MM remains understudied with only a single report on the topic. With improved survival, increasing numbers of older MM patients are now considered for CAR-T [96]. Parker et al. reported that 77% of patients with R/R MM undergoing CAR-T had myopenia. Myopenia was associated with higher rates of neurotoxicity (26% vs. 0%, *p* = 0.04), prolonged hospitalization (≥9 days: 51% vs. 21%, *p* = 0.05), and need for post-treatment rehabilitation or supportive care [97].

Taken together, these data demonstrate that sarcopenia and myopenia are highly prevalent across the MM disease continuum, but variability in definitions and measurement approaches limits comparability across studies. Myosteatosis may be more relevant in this population compared to myopenia particularly in the transplant-eligible MM population. More studies including measure of muscle strength are needed.

## 5. Proposed Mechanisms of Sarcopenia Development and Pathogenesis in Older Adults with Blood Cancers

Traditional mechanisms underlying sarcopenia have centered around perturbations to satellite cells, mitochondrial dysfunction, myosteatosis, altered protein turnover, and denervation [98,99,100,101]. These factors have been largely attributed to malnutrition, insulin resistance, hypogonadism, inactivity, and inflammation, which can each independently contribute to sarcopenia but often converge to drive atrophy and function loss with aging [102,103]. Further, each of these biological and behavioral mechanisms may be amplified with a cancer diagnosis which coalesce to exacerbate sarcopenia (Figure 1).

Malnutrition has long been considered a driver of sarcopenia [104]. Malnutrition impacts between 20 and 70% of older adult blood cancer patients at diagnosis with roughly 1/3 of these patients characterized as obese [105,106]. While the impact of obesity on survivorship remains contentious (i.e., Obesity Paradox), there is considerable evidence to suggest that insulin resistance and myosteatosis negatively impact skeletal muscle mass and function. Sarcopenic obesity is known to be a particularly high-risk body composition phenotype [98,107]. Treatments for blood cancers can induce nausea and vomiting, further worsening malnutrition and insufficient protein intake [104,105].

Beyond malnutrition, several other mechanisms contribute to sarcopenia development among older adults with blood cancers. While there is inherent complexity in the etiology and progression of blood cancers, there is relative homogeneity related to anemia and immune dysregulation leading to fatigue and chronic unchecked inflammation. Fatigue-induced inactivity and muscle disuse directly contributes to skeletal muscle mass and strength loss, most notably through anabolic suppression and reduced mechanical loading [108,109]. Inflammation impacts skeletal muscle mass and function through more diverse means [110]. Increased inflammatory cytokines, namely cytokine families of IL-6, TNFα, and TGFβ, can induce mitochondrial dysfunction and increase proteasomal activity leading to sarcopenia and/or cachexia [111]. Cancer cachexia is prevalent both at diagnosis and as patients require additional lines of therapy further contributing to sarcopenia development [24,112,113,114]. Adipokines, proteins secreted from adipose tissue that activate signaling in peripheral tissues, are implicated in sarcopenia both from aging and cachexia. Key adipokines include resistin, a driver of insulin resistance, and the food intake regulators leptin and adiponectin. These later two adipokines play countering roles; adiponectin has been shown to stimulate food intake while leptin suppresses appetite, reduces food intake, and reduces weight. However, in a meta-analysis assessing the impact of adiponectin on sarcopenia in community-dwelling older adults without cancer, those with sarcopenia had significantly higher levels of adiponectin than controls [115]. Similarly, Smiechowska et al. demonstrated a decrease in plasma leptin levels with no changes in adiponectin and resistin in patients with cachexia in a variety of solid tumors [116]. In this study, low leptin levels were associated with anorexia and increased IL-6. The exact roles of adipokines in the development of sarcopenia, both due to aging and cachexia, remain to be fully understood. Several anti-cancer therapies also have been demonstrated to induce increased mitochondrial dysfunction and protein degradation directly [13,117,118,119]. Corticosteroids are frequently utilized for the treatment of hematologic malignancies (lymphomas, MM, ALL) and as supportive-care medications. Steroid-induced myopathy is a well-documented side effect further contributing to sarcopenia. While more recent work has investigated the role of particular immune cell subsets in muscle mass maintenance with cancer and its treatments [120], to date, these studies have not extended beyond solid tumors. Finally, there is emerging evidence for the role of blood cancer-induced dysbiosis at time of diagnosis on incident cachexia and sarcopenia [113].

**Figure 3:**
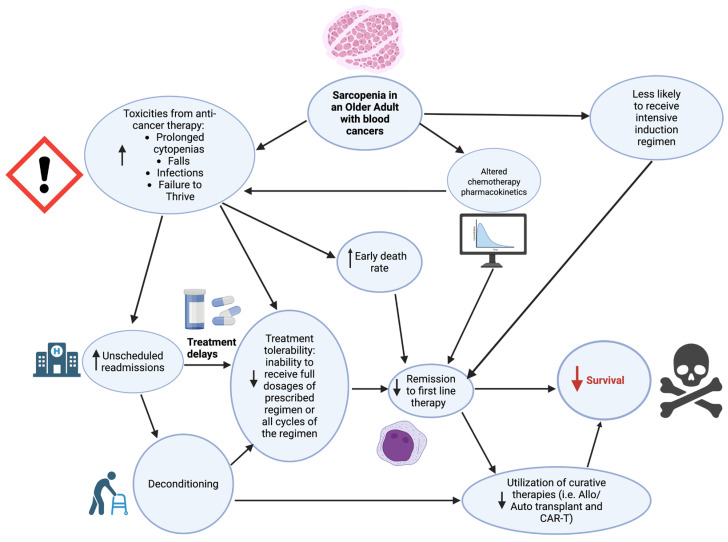
Proposed mechanisms for the influence of sarcopenia on poor survival among older adults with blood cancers. ↑ indicates increase; ↓ indicates decrease. Created in BioRender. Yates, S. (2026) https://BioRender.com/z76b34w.

Hypothesized explanations for why sarcopenic older adults with blood cancers fare worse than their non-sarcopenic counterparts have focused on altered chemotherapeutic drug metabolism and treatment tolerability (Figure 3).

Dosing of most anti-cancer drugs is based only on height and weight to calculate body surface area (BSA), for which the recommended milligrams per meter squared are derived from clinical trials assessing dose-limiting toxicities. Given the muscle mass decline of sarcopenia often co-occurs with increases in fat mass, the pharmacokinetics of anti-cancer drugs are likely altered. Hydrophilic drugs may have increased volume of distribution and half-life, thereby leading to a risk of accumulation and re-release. Hydrophobic drugs may have decreased volume of distribution and increased free fraction-bound drug leading to overdose [121]. In a pan-cancer systematic review of older adults receiving anti-cancer treatments including anthracyclines, cyclophosphamide, and tyrosine kinase inhibitors, those with low muscle mass had higher toxicity rates compared to those with normal muscle mass [121]. In light of these data, a recent randomized trial in patients with stage III colon cancer utilized lean body mass dosing of adjuvant oxaliplatin aiming to decrease incident neuropathy. Investigators found superior rates of Grade ≥ 2 peripheral neuropathy without compromising disease control using this approach compared to standard of care dosing by BSA [122].

Higher toxicity rates exert effects on long-term survival via directly leading to early death and decreasing the ability of the patient to receive the full course of the prescribed regimen (treatment tolerability). Additionally, treatment toxicities often lead to unscheduled readmissions where patient fitness may further decline due to prolonged inactivity. In the end, this loss of fitness leads to loss of eligibility for potentially curative treatments. Finally, skeletal muscle serves essential functions beyond sustaining locomotion. Skeletal muscle is a key metabolic organ for insulin-mediated glucose update, and its secretome (i.e., myokines) has been demonstrated to impact numerous organ systems [123,124]. Taken together, the mechanisms of sarcopenia pathogenesis are, to date, complex and poorly understood, though likely with pharmacologic, behavioral, and metabolic components, each deserving further validation and investigation.

## 6. Intervening on Sarcopenia

### 6.1. Fitness Assessment at Diagnosis

**Figure 4:**
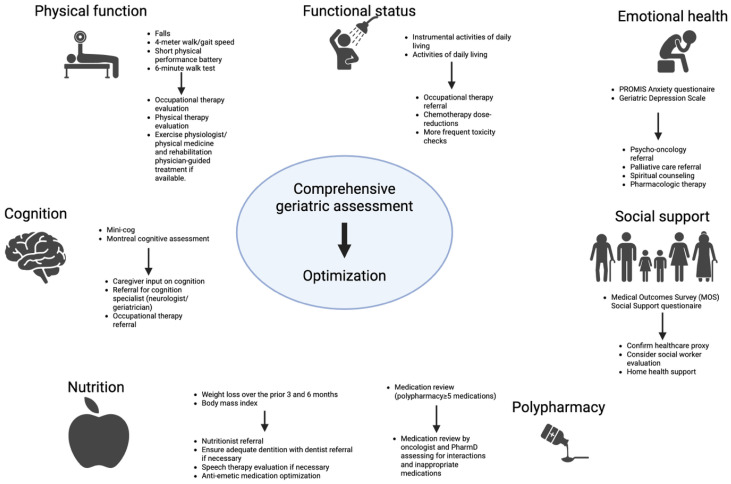
The comprehensive geriatric assessment in older adults with cancer. A CGA assesses then optimizes 7 domains of an older adult’s health both at diagnosis and longitudinally throughout an older adult’s care for cancer. Various CGAs exist with inclusion of varying degrees of the tools noted below. Optimization strategies listed here reflect the guideline (Practical Geriatric Assessment) from ASCO with endorsements from the Cancer and Aging Research Group and the International Society of Geriatric Oncology. Created in BioRender. Yates, S. (2026) https://BioRender.com/dq7b8mo.

Sarcopenia is not an isolated medical condition in older adults with blood cancers. For example, in the HOVON 139/GiVE trial 61% of CLL patients presented with ≥2 impairments on the CGA prior to initiating any anti-leukemic therapy. Therefore, a systematic, holistic approach to assess an older adult’s health is necessary to personalize a sarcopenia intervention to an individual patient. This ought to be done utilizing a CGA, a battery of testing evaluating multiple domains of an older adult’s health: physical function, functional status, comorbidity burden, nutrition/body composition, cognition, mental health, social support, and polypharmacy (Figure 4).

Importantly, the goal of the CGA is to not only identify limitations within a domain but intervene on that domain to improve treatment outcomes. Indeed, among older adults with blood cancers receiving alloSCT and CAR-T, utilization of a CGA-guided optimization program has been shown to improve NRM [125,126]. The CGA is the gold-standard for fitness assessment and optimization prior to systemic therapy for older adults with blood cancers and, therefore, recommended by the International Society of Geriatric Oncology, American Society of Clinical Oncology (ASCO), and European LeukemiaNet [26,127,128,129].

### 6.2. Protein Supplementation and Resistance Training Exercise

The primary aim of sarcopenia management is to maintain or improve muscle mass and strength, thereby improving a patient’s physical function. Among older adults without cancer, this has translated into improvement in mobility disability in randomized trials [130,131]. To date, no trials have been conducted with the primary endpoint of sarcopenia among older adults with blood cancers. The mainstay of sarcopenia treatment in older adults without cancer is protein supplementation and resistance exercise training. The European Society of Parenteral and Enteral Nutrition guidelines for nutrition in patients with cancer recommends 1.0–1.5 g/kg/day of protein, though this is not specific to those who are sarcopenic or older adults [132]. Additionally, the authors note that upwards of 2.0 g/kg/day may be necessary, though this is not sufficiently supported yet by the literature. A position paper from the Society of Sarcopenia, Cachexia and Wasting Disorders also endorsed up to 2.0 g/kg/day “in those with evidence of a pro-inflammatory/catabolic state” such as cancer cachexia [133]. The ideal timing and source of dietary protein is also an ongoing debate [134]. Guidelines for the approach to meet these protein goals are outlined by ESPEN [132]. Oral nutrition, with or without oral nutritional support, ought to be utilized first followed by enteral nutrition. Perenteral nutrition is utilized sparingly in hematologic malignancies due to fear of infectious complications in those who are neutropenic. Other nutritional interventions under investigation include fish oil, eicosapentaenoic acid (EPA), oligopeptides (creatine, carnitine), leucine and its derivative hydroxymethyl butyrate, and various nutraceuticals [135,136]. EPA may be taken alone or found as a component of fish oil. EPA/fish oil is an anti-inflammatory substance with evidence to support improvement in appetite and dietary intake, particularly in those with cachexia. EPA/fish oil has shown mixed results in preventing muscle loss in patients with solid tumors. Combining EPA/fish oil with protein supplementation may be a compelling nutritional therapy given the different mechanisms these modalities utilize to treat sarcopenia. The utility of oligopeptides and nutraceuticals to treat sarcopenia are, thus far, mixed and are extensively reviewed elsewhere [135,136].

Resistance exercise is effective in improving both muscle mass and strength in healthy older adults with and without sarcopenia [137,138]. Mixed modality programs including aerobic and balance exercise have also been shown to be effective [130]. A 2019 Cochrane review of aerobic exercise among patients with blood cancers found 18 eligible studies suggesting aerobic exercise interventions may improve fatigue but were inconclusive as to whether they would improve physical function. No studies were specific to older adults and there was heterogeneity as to whether the studies were conducted during active chemotherapy or in the survivorship setting [139]. Klepin et al. conducted a randomized trial among older adults with AML utilizing a symptom-adapted, inpatient physical activity intervention in comparison to standard-of-care supportive care. The intervention was feasible (primary endpoint) and suggested physical performance may be maintained or improved [140]. Feasibility of an at-home, mobile health exercise program using resistance and aerobic training has also been established among older adults with AML, with an efficacy trial ongoing [141,142]. Among older adults with lymphoma and MM undergoing chemotherapy, feasibility of an Otago Exercise Program, emphasizing resistance, balance, and aerobic training, has also been established [143]. Furthermore, the REAL-FITNESS trial randomized newly diagnosed patients with MM (median age of 67 years) receiving chemotherapy to standard supportive care versus personal-trainer guided exercise according to WHO recommendations: 150 min of moderate intensity aerobic exercise weekly plus two, hour long resistance training sessions weekly. At the end of the 3-month intervention, patients randomized to the exercise arm showed improvement in physical performance, muscle strength, fatigue, and quality of life (QOL) [144]. Finally, several trials have assessed the impact of exercise on maintenance or improvement in physical function and/or functional status after alloHSCT or autoHSCT [145,146,147,148]. A supervised, in-person 12-week exercise randomized controlled trial conducted among those ≤6 months post-transplant showed improvement in 6-min walk test (6-MWT) compared with standard care [147]. A recent randomized trial of a 6-week telehealth exercise and mindfulness training conducted among those at least 6 months out from autoHSCT or alloHSCT led to a clinically meaningful change in 6-MWT at 3 months and 12 months [146]. To date, no trials utilizing exercise have been conducted exclusively among older adults post-transplant.

### 6.3. Cancer Cachexia

There are currently no treatments for cancer cachexia approved by the USA’s Food and Drug Administration (FDA) or the European Medical Agency (EMA). Only one drug, anamorelin (discussed below), is approved in Japan [149]. Furthermore, all trials of medications discussed in this section have excluded patients with blood cancers, with no older adult specific trials conducted either. Investigational therapeutics of cachexia broadly target one of three mechanisms: appetite modulation, inflammation blockage, and metabolic derangements [12]. Agents impacting appetite include megestrol acetate [150], cannabinoids [151], growth differentiation factor 15 (GDF-15) antagonists (ponsegromab) [152], ghrelin mimetics (anamorelin) [153], and olanzapine [154]. In the ROMANA 1 and 2 Phase 3 trials, anamorelin improved lean body mass, appetite, and weight, but not grip strength or QOL among patients with non-small cell lung cancer [153]. However, a similar multi-center study in Japan did show improvement in QOL leading to its approval [155]. Ponsegromab is a monoclonal antibody against GDF-15 recently shown to improve weight, physical activity, QOL, muscle mass, and appetite compared to placebo in a randomized Phase II trial of patients with advanced stage solid tumors [152]. Ponsegromab acts on the brainstem to modulate appetite and likely has an anti-inflammatory effect systemically [156]. The antipsychotic drug olanzapine, known for its side effect of appetite stimulation and anti-emetic properties, is now recommend by ASCO at low dose (2.5 mg daily) based on the results of a recent randomized trial showing improvement in weight, appetite, and QOL in patients with advanced solid tumors compared to placebo [154,157].

Anti-inflammatory agents trialed thus far include IL-6 inhibitors (Clazakizumab [158]), TNF-alpha inhibitors (Infliximab [159]), non-steroidal anti-inflammatory drugs (NSAIDS) [160], and corticosteroids [150,161]. The Multimodal Exercise Nutrition Anti-inflammatory Cachexia (MENAC) trial was an international, randomized Phase III trial utilizing exercise, nutrition, and NSAIDs among lung and pancreatic cancer patients receiving standard of care therapy [162]. Early results showed improvement in weight but not muscle mass or strength; the full trial results are awaited [160]. Corticosteroids are recommended as an option for cachexia management by ASCO and the European Society of Medical Oncology, given for a short period only (2–3 weeks). Toxicities including myopathy, hyperglycemia, and psychiatric toxicities can be prohibitive with longer term use with benefit only to appetite [163,164]. JAK2 inhibitors, including Ruxolitinib, are frequently utilized in the treatment of multiple blood cancers and thus present a compelling anti-cachectic agent in this population. The utilization of this agent to target cachexia is still in the Phase I setting (NCT04906746).

Finally, anabolic agents including selective androgen receptor modulators [165] and beta-adrenergic receptor blockers (Beta-blockers) [166] have been evaluated to mitigate metabolic derangements found in cachexia. These agents have not yet been successful in the Phase III setting. Enobosarm showed improvement in muscle mass and function in Phase II trials [165] though Phase III studies POWER I and II appeared negative with full results not yet published (NCT01355484 and NCT01355497) nearly a decade out from completion. S-pindolol, a non-specific Beta1/Beta2 antagonist, showed improvement in muscle mass and strength compared to placebo in the randomized Phase II ACT-ONE trial among lung and colorectal cancer patients. Phase III trials are anticipated.

## 7. Future Directions

Age is the single biggest risk factor for both sarcopenia and hematologic malignancies [7,19]. As the world’s population ages [167], it is of pressing importance for health care providers and health systems to understand the growing burden of sarcopenia and its implications for older adults with hematologic malignancies. However, despite having an ICD-10-CM diagnosis code (M62.84) assigned to sarcopenia in 2016, there remains no recognized, unified criteria to date [168,169]. The development of the GLIS and its conceptual definition of sarcopenia is a major step forward in this goal. The operational definition to be utilized in the clinical and research setting is eagerly awaited [37]. The absence of unified consensus criteria has led to a severe lack of recognition of sarcopenia among healthcare providers in a variety of disciplines [170]. A recent study assessing the discrepancy between utilization of the ICD-10 code for sarcopenia with myopenia on CT scan found massive discordance among ~18,000 patients; 28.5% of patients had myopenia, yet the ICD code was only utilized in 0.05% of the cohort [171]. The diagnosis of cancer cachexia suffers from similar limitations, likely impacting the lack of recognition of sarcopenia [172]. The lack of a consensus definition for sarcopenia has also led to inaction from international regulatory bodies to recognize sarcopenia as a treatable condition, thereby providing limited incentive for drug development [169]. Thus, the work of the GLIS is an essential step forward for the field.

A historical limitation to obtain the measure of muscle mass necessary for sarcopenia was time for radiologist assessment. However, this is increasingly being remedied by the utilization of artificial intelligence (AI) imaging processing tools [173,174]. To date, these are not standard-of-care and are limited to the research setting. Significant implementation science hurdles will need to be conquered to bridge that gap, but advances in this field are rapid [175,176]. In theory, any CT, MRI, or PET scan utilized in routine disease evaluation for lymphomas and myelomas could have a muscle mass data point available in the imaging report. For leukemias, where CT and MRI are not a standard of care diagnostic component at diagnosis or longitudinally, utilization of BIA will be necessary. The measure of muscle mass ought to be done in conjunction with a CGA. A CGA generally measures muscle strength and captures limitations that may impair an older adult’s ability to undergo sarcopenia optimization.

Sarcopenia may be relevant both as a biomarker of fitness and to track the impact of supportive care interventions. Specifically, by measuring both muscle mass and strength, sarcopenia may serve as an objective biomarker to evaluate the impact of cachexia, nutrition, and physical activity interventions. However, rigorous studies are needed to evaluate and validate the most prognostically relevant cut points for muscle mass and strength within older adults with hematologic malignancies. Additionally, the clinically meaningful improvement in muscle mass and strength must be evaluated for use in interventional trials. The paucity of interventional trials targeting sarcopenia or cancer cachexia among patients with blood cancers is a staggering discrepancy compared to solid tumors. Patients with lymphoma and myeloma receiving CAR-T are a compelling initial population to pursue such interventions. Available data show that myopenia is prevalent and negatively impacts treatment outcomes. Pre-habilitation clinics have shown early success in improving NRM in older adults receiving CAR-T [125]. Pilot studies ought to incorporate a high-protein diet (1.5–2 g/kg), resistance exercise, and anti-cachectic agents for those with concomitant cachexia. Combining EPA/fish oil with protein supplementation may be a compelling nutritional therapy given the different mechanism these modalities utilize to treat sarcopenia. Trials would benefit from evaluation of outcomes related to feasibility, treatment tolerance, and QOL. Qualitative approaches to elicit patient and caregiver feedback on the intervention ought to be incorporated. Cost-effectiveness analysis of incorporating sarcopenia assessment and interventions into usual care pathways are also needed. Finally, biobanking samples (blood, stool, and muscle) will be essential to continue to understand the unique biology underpinning sarcopenia’s development and adverse effects on older adults with blood cancers.

## 8. Conclusions

Sarcopenia is a prevalent and prognostically relevant biomarker of patient fitness among older adults with blood cancers. Unifying the diagnostic criteria for sarcopenia is essential and forthcoming through the work of the GLIS. Measurement of both muscle mass and strength is required by the GLIS definition for the diagnosis of sarcopenia. Most studies in oncology utilize only a measure of muscle mass (myopenia) in their definition of sarcopenia reflecting outdated definitions of the condition. Educational initiatives are greatly needed in oncology to educate clinicians on how to diagnose sarcopenia and the impact it may have on their patient’s treatment outcome. Sarcopenia interventions should begin with a holistic assessment of an older adult’s health via a CGA. Interventional trials to improve sarcopenia using CGA-guided optimization strategies incorporating a high-protein diet, resistance exercise, and anti-cachectic agents are eagerly awaited.

## Figures and Tables

**Table 1:** Criteria recommended for defining sarcopenia by international consensus groups. The definitions of the EWGSOP, SDOC, and AWGS are presented as they reflect consensus from Europe, USA, and Asia, respectively, prior to the GLIS definition.

Organization	Year	Definition Components	Muscle Mass	Muscle Strength	Physical Performance
European Working Group on Sarcopenia in Older People2 (EWGSOP2) [35], also endorsed by the Australian and New Zealand Society for Frailty Research (ANZSSFR) [36]	2019	Low muscle strength (primary); confirmed by low muscle mass; severity assessed by physical performance	ALMI: ALM/height^2^: <7.0 kg/m^2^ (men), <5.5 kg/m^2^ (women)	Handgrip strength: <27 kg (men), <16 kg (women)	Gait speed < 0.8 m/s SPPB ≤ 8 TUG ≥ 20 s 400 m walk test ≥ 6 min 5-time chair stand > 15 s
Asian Working Group for Sarcopenia (AWGS) [34]	2019	Low muscle mass + low muscle strength and/or low physical performance	ALMI: ALM/height^2^: <7.0 kg/m^2^ (men, BIA or DXA), <5.4 kg/m^2^ (women, using DXA); <5.7 kg/m^2^ (women, using BIA)	Handgrip strength: <28 kg (men), <18 kg (women)	Gait speed < 1.0 m/s SPPB ≤ 9 5-time chair stand ≥ 12 s
Sarcopenia Definition and Outcomes Consortium (SDOC) [33]	2020	Low muscle strength and low physical performance	Not used	Hand grip strength: <35.5 kg (men), <20 kg (women)	Gait speed < 1.0 m/s
Global Leadership in Sarcopenia [37]	2024	Low muscle mass + low muscle strength	Cut offs not yet determined	Cut offs not yet determined	Used as an outcome, not diagnostic criteria

Abbreviations: ALM, appendicular lean mass; ALMI, appendicular lean mass index (=ALM/height^2^, where height is in meters); AWGS, Asian Working Group for Sarcopenia; BIA, bio-electrical impedance analysis; DXA, dual-energy X-ray absorptiometry; EWGSOP2, European Working Group on Sarcopenia in Older People 2; SDOC, Sarcopenia Definitions and Outcomes Consortium; SPPB, short physical performance battery; TUG, timed up-and-go.

**Table 2:** Studies assessing sarcopenia among patients with hematologic malignancies.

Study	Hematologic Malignancy	Number of Patients	Median Age; Range (Years), % Older Adults	Treatment Regimen	Definition of Sarcopenia or Myopenia	Mass Measurement	Strength Measurement	Sarcopenia or Myopenia Prevalence (%)	Outcomes and Their Association with Sarcopenia or Myopenia
Nakamura et al. [47]	AML	90	54 (18–84), 49%	Intensive chemotherapy (73%), less-intensive chemotherapy (23%)	^+^SMI at L3 < 48.4 cm^2^/m^2^ (males), SMI at L3 < 33.5 cm^2^/m^2^ (females)	SMI at L3 by CT	None	Whole cohort (43%), Older adult subgroup (41%)	Associated with OS, EFS, DFS in whole cohort and older adult subgroup
Jung et al. [48]	AML	96	58 (18–84), 46.9%	Intensive chemotherapy (79%), less-intensive chemotherapy (21%)	^+^SMI at L1 < 40.79 cm^2^/ m^2^ (males), SMI at L1 < 31.6 cm^2^/m^2^ (female)	SMI at L1 by PET-CT or CT	None	Whole cohort (58%), older adult subgroup (41.7%)	Associated with OS and TRM
Sun et al. [49]	AML	227	64 (24–87), 67%	Intensive chemotherapy (25%), less intensive chemotherapy (75%)	EWGSOP2 criteria [35]: ASMI < 7.0 kg/m^2^ for men, ASMI < 5.5 kg/m^2^ for women	BIA-derived ASMI	None	Whole cohort (18%), older adult subgroup (21%)	Associated with OS, DFS
Fang et al. [50]	AML	219	64 (53–70), 62.5%	Intensive chemotherapy (20%), less intensive chemotherapy (80%)	EWGSOP2 criteria [35]: ASMI < 7.0 kg/m^2^ for men, ASMI < 5.5 kg/m^2^ for women	BIA-derived ASMI	None	Whole cohort (16%), older adult subgroup (NR)	Associated with OS
Qin et al. [51]	AML	69	53 (44.5–61.0), NR	Intensive chemotherapy (100%)	AWGS criteria: [34]: Mass: <5.7 kg/m^2^ in women and <7.0 kg/m^2^ in men. Strength: <18 kg in women and <28 kg in men	BIA-derived ASMI	Grip strength	Whole cohort (14.5%), older adult subgroup (NR)	None assessed
Yates et al. [24]	AML	79	73 (60–93), 100%	Intensive chemotherapy (8%), Less-intensive chemotherapy (83%), unable to initiate induction due to fatality (9%)	SMI at L1 < 25.9 cm^2^/m^2^ for females, <34.6 cm^2^/m^2^ for males [52] Grip strength adjusted for BMI [53]	CT-derived SMI at L1	Grip strength	Whole cohort was older adult (24%)	Associated with early death, treatment tolerability, readmissions. Numerically associated with OS (13.8 months vs. 2.9 months; *p* = 0.1).
Armenian et al. [54]	AML, ALL, or MDS	859	51 (18–74), NR	AlloHSCT	Males: SMI at L3 < 43 cm^2^/m^2^ (BMI < 25 kg/m^2^) or SMI < 53 cm^2^/m^2^ (BMI ^3^25 kg/m^2^) [55] Females: SMI at L3 < 41 cm^2^/m^2^ (regardless of BMI)	CT-derived SMI at L3	None	Whole cohort (33.7%), older adult subgroup (NR)	Associated with OS and early death
Ando et al. [56]	AML, MDS	125	47 (16–65), NR	AlloHSCT	SMI at L3 < 50.9 cm^2^/m^2^ (males), SMI at L3 < 48.4 cm^2^/m^2^ (females) [46]	CT-derived SMI at L3	None	Whole cohort (41.6%), older adult subgroup (NR)	Associated with OS and early death
van der Straten et al. [21]	CLL	67	71 (68–75), 100%	Venetoclax + Obinutuzumab	EWGSOP2 criteria [35]: ^+^SMI at L3 < 41.6 cm^2^/m^2^ (males) and 32.0 cm^2^ /m^2^ (females) Grip Strength: <27 g (males), <16 kg (females) [35]	CT-derived SMI at L3	Grip strength	Whole cohort older adult (13%)	None assessed
Rios Olais et al. [57]	ALL	90	37 (18–72), NR	Intensive chemotherapy (60%), less intensive chemotherapy (40%)	SMI at L3 < 55 cm^2^/m^2^ (men), SMI at L3 < 39 cm^2^/m^2^ in women [46]	CT-derived SMI at L3	None	Whole cohort (76.7%), older adult subgroup (NR)	Associated with OS
Nakamura et al. [58]	DLBCL	207	67 (19–86), 71%	R-CHOP (56%), R-THP-COP (44%)	SMI at L3 < 47.1 cm^2^/m^2^ in males and <34.4 cm^2^/m^2^ in females [9]	CT-derived SMI at L3	None	Whole cohort (56%), older adult subgroup (63%)	Associated with OS, PFS
Lanic et al. [59]	DLBCL	82	80.2 (mean) (70–95), 100%	R-CHOP (55%), R-miniCHOP (45%)	^+^SMI at L3 < 55.8 cm^2^/ m^2^ (males) and 38.9 cm^2^/m^2^ (females)	CT-derived SMI at L3	None	Whole cohort older adult (55%)	Associated with OS, PFS
Pénichoux et al. [60]	DLBCL	97	78.4 (mean) (70–92), 100%	R-CHOP (58%), R-miniCHOP (42%)	^+^SMI at L3 < 55.8 cm^2^/ m^2^ (males) and 38.9 cm^2^/m^2^ (females)	CT-derived SMI at L3	None	Whole cohort older adult (56%)	Not associated with OS or PFS
Chu et al. [61]	DLBCL	224	62, (21–88), NR	R-CHOP (100%)	^+^SMD: BMI < 25 kg/m^2^: 33 HU BMI^3^25 kg/m^2^: 40 HU	Mean muscle attenuation at L3	None	Whole cohort (51.7%), older adult subgroup (NR)	Associated with OS
Zilioli et al. [62]	Hodgkin Lymphoma	154	71, (NR), 100%	ABVD (76%), other chemotherapy (21%)	^+^SMI at L3 < 55.8 cm^2^/ m^2^ (males) and 38.9 cm^2^/m^2^ (females); <47.1 cm^2^/m^2^ (males) and <34.4 cm^2^/m^2^ (females) [9]	CT-derived SMI at L3	None	Whole cohort was older adult (73%)	Not associated with OS and PFS
Albano et al. [63]	Mantle Cell Lymphoma	53	72.7 (mean) (66–82), 100%	R-BAC, R-CHOP, R-hyperCVAD (percentages not reported)	^+^SMI at L3 < 53 cm^2^/ m^2^ (males) and 45.6 cm^2^/m^2^ (females)	CT-derived SMI at L3	None	Whole cohort was older adult (60%)	Associated with PFS
Armenian et al. [64]	Hodgkin and Non-Hodgkin Lymphoma	320	53.3 (18.5–78.1), NR	autoHSCT (100%)	SMI male: <43 cm^2^/m^2^ (BMI < 25 kg/m^2^) or <53 cm^2^/m^2^ (BMI ≥ 25 kg/m^2^) and female: <41 cm^2^/m^2^ (regardless of BMI) [55]	CT-derived SMI at L3	None	Whole cohort (34%), older adult subgroup (NR)	Associated with length of hospitalization, ICU admission, unplanned readmission, OS
Lin et al. [65]	Hodgkin and Non-Hodgkin Lymphoma	146	60.7 (50−78.7), NR	alloHSCT (100%)	SMI male: <43 cm^2^/m^2^ (BMI < 25 kg/m^2^) or <53 cm^2^/m^2^ (BMI ≥ 25 kg/m^2^) and female: <41 cm^2^/m^2^ (regardless of BMI) [55]	CT-derived SMI at L3	None	Whole cohort (55%), older adult subgroup (NR)	Associated with OS, PFS, non-relapse mortality
Rejeski et al. [66]	DLBCL, transformed indolent lymphoma	106	64 (19–83), NR	CAR-T: axicabtagene ciloleucel (64%), tisagenlecleucel (36%)	^+^SMI < 34.5 (cm^2^/m^2^)	CT-derived SMI at L3	None	Whole cohort: 10%, older adult subgroup (NR)	Associated with PFS and OS
Jhaveri et al. [67]	DLBCL, transformed indolent lymphoma	83	66 (36–86), NR	CAR-T: axicabtagene ciloleucel (76%), tisagenlecleucel (21.6%), lisocabtagene maraleucel (2.4%)	SMI < 52.4 cm^2^/m^2^ for males and <38.5 cm^2^/m^2^ for females [68]	CT-derived SMI at L3	None	Whole cohort (53%), older adult subgroup (NR)	Associated with non-relapse mortality, PFS, OS
Williams et al. [69]	MM	142	62.4 (38.2–78.7), NR	autoHSCT	^+^≤80% high-density muscle. High-density (>+29 HU)	Percentage of high-density muscle within the psoas muscle at L3 on CT or PET-CT.	None	Whole cohort (51%), older adult subgroup (NR)	Associated with early post-transplant complications (100 days) and PFS. Not associated with OS.
Abdallah et al. [70]	MM	341	65 (59–72), 83%	Induction +/− autoHSCT	^+^Various cutoffs for SMI at L3 1- < sex-specific median 2- < 52.4 cm^2^/m^2^ in men and <38.5 cm^2^/m^2^ in women 3- < 55 cm^2^/m^2^ in men and <39 cm^2^/m^2^ in women 4- ≤ 43 cm^2^/m^2^ if BMI is <25 kg/m^2^ or ≤53 cm^2^/m^2^ if BMI is ≥25 in men and <41 cm^2^/m^2^ in women irrespective of BMI	PET/CT-derived SMI at L3	None	Whole cohort (46–56%) depending on the cutoff used, older adult subgroup (NR)	Associated with PFS, OS
Ordonez et al. [71]	MM	129	Mean ± SD, (59.17 ± 7.2), NR	autoHSCT	Males: SMI at L1 < 43 cm^2^/m^2^ (BMI < 25 kg/m^2^) or SMI < 53 cm^2^/m^2^ (BMI ^3^25 kg/m^2^) [55] Females: SMI at L1 < 41 cm^2^/m^2^ (regardless of BMI)	CT-derived SMI at L1	None	Whole cohort (86.6%), older adult subgroup (NR)	Not associated with early post-transplant adverse events (30 days), PFS or OS
Survov et al. [72]	MM	123	Mean ± SD (57.8 ± 7.6), NR	autoHSCT	Males: SMI at L3 < 52.4 cm^2^/m^2^ [68] Females: SMI at L3 < 38.5 cm^2^/m^2^	CT-derived SMI at L3	None	Whole cohort (33.3%), older adult subgroup (NR)	Not associated with PFS or OS
Annibali et al. [73]	MM	68	58 (38–70), 33%	autoHSCT	Males: SMI at L3 < 52.4 cm^2^/m^2^ [68] Females: SMI at L3 < 38.5 cm^2^/m^2^	CT-derived SMI at L3	None	Whole cohort 54%, older adult subgroup (69%)	Not associated with PFS or OS
Park et al. [74]	MM	190	Mean ± SD (55.9 ± 7.0), NR	Induction + utoHSCT	Lower than 20th percentile of sex-specific quintile [69]: 92 cm^2^/m^2^ for males and 81 cm^2^/m^2^ for females.	CT-derived Paraspinal muscle index (PMI) at T12	None	Whole cohort 20%, older adult subgroup (NR)	Associated with PFS, OS, TRM
Takeoka et al. [75]	MM	56	71 (NR), NR	Induction +/− autoHSCT	Males: SMI at L3 < 43 cm^2^/m^2^ (BMI < 25 kg/m^2^) or SMI < 53 cm^2^/m^2^ (BMI ^3^25 kg/m^2^) [55] Females: SMI at L3 < 41 cm^2^/m^2^ (regardless of BMI)	CT-derived SMI at L3	None	Whole cohort 66%, older adult subgroup (NR)	Not associated with OS
Nandakumar et al. [76]	MM	322	66 (37–95), NR	Induction + autoHSCT	Male: SMI at L3 < 55 cm^2^/m^2^ [46] Female: SMI at L3 <39 cm^2^/m^2^	CT-derived SMI at L3	None	Whole cohort 53%, older adult subgroup (NR)	Associated with OS
Diallo et al. [77]	MM	226	65 (29–89), NR	Induction chemotherapy +/− autoHSCT	Males: SMI at L3 < 43 cm^2^/m^2^ (BMI < 25 kg/m^2^) or SMI < 53 cm^2^/m^2^ (BMI ^3^25 kg/m^2^) [55] Females: SMI at L3 < 41 cm^2^/m^2^ (regardless of BMI)	CT-derived SMI at L3	None	Whole cohort 51%, older adult subgroup (NR)	Associated with OS and PFS
Tagliafico et al. [78]	MM	74	61 (36–89), NR	Induction chemotherapy + autoHSCT/alloHSCT	SMI < 41 cm^2^/m^2^ in males and females [79]	CT-derived SMI at L3	None	Whole cohort (24%), older adult subgroup (NR)	Not associated with OS
Stege et al. [80]	MM	220	78 (65–92), 100%	Induction Chemotherapy without autoHSCT	EWGSOP2 criteria [35] ^+^SMI at L3 < 41.6 cm^2^/m^2^ (males) and 32.0 cm^2^ /m^2^ (females) Grip Strength: <27 g (males), <16 kg (females)	CT-derived SMI at L3	Grip strength	Whole cohort older adult (15%)	Myopenia, but not sarcopenia, associated with treatment discontinuation and OS

ALL, acute lymphocytic leukemia; AlloHSCT, allogeneic stem cell transplant; AML, acute myeloid leukemia; ASMI, appendicular skeletal muscle index; AutoHSCT, autologous stem cell transplant; BIA, bioelectrical impedance analysis; CAR-T, chimeric antigen receptor T-cell therapy; CLL, chronic lymphocytic leukemia; CT, computed tomography; DFS, disease-free survival; DLBCL, diffuse large B-cell lymphoma; EFS, event-free survival; HU, Hounsfield Unit; MDS, myelodysplastic syndrome; OS, overall survival; MM, multiple myeloma; PET, positron emission tomography; PFS, progression-free survival; SMD, skeletal muscle density; SMI, skeletal muscle index; TRM, treatment-related mortality. ^+^Cut point for low mass internally derived in the individual study.

## Data Availability

No new data were created or analyzed in this study. Data sharing is not applicable to this article.

## Abbreviations

The following abbreviations are used in this manuscript:

ALL	Acute lymphoblastic leukemia
AML	Acute myelocytic leukemia
AlloHSCT	Allogeneic stem cell transplant
ASCO	American Society of Clinical Oncology
ALM	Appendicular Lean Mass
ASMM	Appendicular Skeletal Muscle Mass
AWGOS	Asian Working Group on Sarcopenia
ANZSSFR	Australian and New Zealand Society for Frailty Research
AutoHSCT	Autologous stem cell transplant
BIA	Bioelectrical impedance analysis
BMI	Body mass index
BSA	Body surface area
CAR-T	Chimeric antigen receptor T-cell therapy
CLL	Chronic lymphocytic leukemia
CGA	Comprehensive geriatric assessment
CT	Computed tomography
DLBCL	Diffuse large B-cell lymphoma
DFS	Disease-free survival
DEXA	Dual-energy X-ray absorptiometry
EPA	Eicosapentaenoic acid
EMA	European Medical Agency
EWGSOP	European Working Group on Sarcopenia in Older People
EFS	Event-free survival
FL	Follicular lymphoma
FDA	Food and Drug Administration
FNIH	Foundation of National Institutes of Health
GLIS	Global Leadership in Sarcopenia
GDF-15	Growth differentiation factor 15
HR	Hazard ratio
HL	Hodgkin’s lymphoma
HU	Hounsfield Unit
MM	Multiple myeloma
MDS	Myelodysplastic syndrome
nHL	Non-Hodgkin’s lymphoma
OS	Overall survival
PET	Positron emission tomography
QOL	Quality of life
SDOC	Sarcopenia Definitions and Outcomes Consortium
SMD	Skeletal muscle density
SMI	Skeletal muscle index
TRM	Treatment-related mortality
